# The use of participatory systems mapping as a research method in the context of non-communicable diseases and risk factors: a scoping review

**DOI:** 10.1186/s12961-023-01020-7

**Published:** 2023-07-06

**Authors:** Amber van den Akker, Alice Fabbri, Dima I. Alardah, Anna B. Gilmore, Harry Rutter

**Affiliations:** University of Bath, Bath, United Kingdom

**Keywords:** Review, Participatory systems mapping, Non-communicable diseases, Systems thinking, Unhealthy commodities

## Abstract

**Context:**

Participatory systems mapping is increasingly used to gain insight into the complex systems surrounding non-communicable diseases (NCDs) and their risk factors.

**Objectives:**

To identify and synthesize studies that used participatory systems mapping in the context of non-communicable diseases.

**Design:**

Scoping review.

**Eligibility criteria:**

Peer-reviewed studies published between 2000 and 2022.

**Study selection:**

Studies that focused on NCDs and/or related risk factors, and included participants at any stage of their system’s mapping process, were included.

**Categories for analysis:**

The main categories for analysis were: (1) problem definition and goal-setting, (2) participant involvement, (3) structure of the mapping process, (4) validation of the systems map, and (5) evaluation of the mapping process.

**Results:**

We identified 57 studies that used participatory systems mapping for a variety of purposes, including to inform or evaluate policies or interventions and to identify potential leverage points within a system. The number of participants ranged from 6 to 590. While policymakers and professionals were the stakeholder groups most often included, some studies described significant added value from including marginalized communities. There was a general lack of formal evaluation in most studies. However, reported benefits related mostly to individual and group learning, whereas limitations described included a lack of concrete actions following from systems mapping exercises.

**Conclusions:**

Based on the findings of this review, we argue that research using participatory systems mapping would benefit from considering three different but intertwined actions: explicitly considering how different participants and the power imbalances between them may influence the participatory process, considering how the results from a systems mapping exercise may effectively inform policy or translate into action, and including and reporting on evaluation and outcomes of the process, wherever possible.

**Supplementary Information:**

The online version contains supplementary material available at 10.1186/s12961-023-01020-7.

## Introduction

Non-communicable diseases (NCDs) are accountable for 74% of all deaths globally [1]. Policies seeking to reduce the rising burden of NCDs have so far been largely ineffective, leading to calls for better understanding of the complex interplay of risk factors that contribute to them, including the consumption of unhealthy commodities such as tobacco, alcohol and unhealthy foods and beverages, as well as wider socioeconomic, ecological and political determinants [2–4]. In appreciation of this complexity, an increasing number of scholars advocate for a systems approach to address these issues [5–9]. Rather than taking a linear cause and effect approach to a problem, systems approaches emphasize the interconnectedness of different elements and how they interact so that the outcome is greater than the sum of the different parts within the system [10]. Places to intervene in the system, also termed ‘leverage points’, may thus impact not only the direct part of the system in which the intervention is placed, but also the wider system, depending on the scope of the intervention [10]. The World Health Organization (WHO) has recently published a guide to taking a systems thinking approach to NCD prevention, describing the usefulness of this rapidly evolving field of research for the complexity of NCD prevention [11].

The various terminologies used to describe participatory systems mapping or similar processes give some indication of the rapid development in the field from when it was first proposed as an approach by Forrester and Meadows, to it now being increasingly advocated by multiple authors and institutions [10, 12]. Common approaches to participatory systems mapping include ‘causal loop diagrams (CLD)’ [13], ‘collaborative conceptual modelling’ [14], ‘community-based system dynamics (CBSD)’ [15], ‘group model building’ (GMB) [16], and ‘participatory systems mapping’ [17]. We use participatory systems mapping as a term when referring to methods that include stakeholders, usually through one or more workshops, to build a systems overview of a complex problem, usually to support decision-making processes or gain insight into a system of interest [18–20].

Previous reviews on participatory systems mapping approaches by Rouwette et al. and Scott et al. provided an overview of the effectiveness of GMB as one specific approach to participatory systems mapping [21, 22]. Rouwette et al. noted a wide variety in the mapping processes and the extent to which authors assessed their results [21]. While most studies reported increased insights into the problem on the part of participants, fewer than half of the studies Rouwette et al. reviewed reported outcomes at the group or organization level, with only 34 out of 107 reviewed studies considering system mapping more efficient than traditional methods used for similar problems [21]. Similarly, Scott et al. note a general lack of evidence on the contexts in which certain systems mapping tools might be more useful or effective [22].

Our current study builds on the foundation these reviews have laid, although there are important differences. First, we review participatory systems mapping research, including but not limited to GMB. We do so to gain insight into the differences and similarities between different methods that could all be seen as being participatory forms of systems mapping. Second, we focus on research conducted on NCDs and risk factors. As such, our aim in performing this scoping review was to identify and synthesize studies that used participatory systems mapping in the context of NCDs and unhealthy commodities (UCs), here referring to tobacco, alcohol, unhealthy food and sugar-sweetened beverages and gambling. The research aims to present an overview of the purpose and approach to participatory systems mapping in this context, as well as draw out commonalities and differences in how participatory systems mapping is used, with an emphasis on these methods’ participatory components and the lessons learned from using these methods.

## Methods

### Study selection

We conducted a scoping review following the PRISMA Extension for Scoping Reviews (PRISMA-ScR) [23]. The search strategy was developed in consultation with a research librarian. The following databases were searched, which were chosen after consulting a University librarian: SCOPUS, International Bibliography of the Social Sciences (IBSS), Web of Science (all databases) and Pubmed. The following search terms were used:

TITLE-ABS-KEY (NCDs OR ‘noncommunicable disease*’ OR ‘non-communicable disease*’ OR tobacco OR alcohol OR food OR obesity OR drink OR beverage* OR ‘physical activity’ OR ‘physical inactivity’ OR gambl*) AND ‘group model build*’ OR [(community-based OR participatory OR stakeholder*) AND (‘system map*’ OR ‘systems map*’ OR ‘causal loop diagram’ OR ‘causal-loop diagram’)] AND NOT (GIS OR ‘geographic information system’).

We only included papers that were peer-reviewed and published in English between 1 January 2000 and 28 February 2022. Additional sources were identified through hand searching the reference list of included studies.

Titles and abstracts were screened by one reviewer (A.v.d.A.). When inclusion or exclusion was not clear from the title and abstract, the full text was reviewed. Articles where included if they presented empirical research on NCDs or related risk factors, using participatory systems mapping, which was defined as an approach that developed a systems map with input from participants at any stage in the mapping process. We excluded non-empirical articles, including editorials and commentaries. After the first stage of the review process, the full text screening was conducted independently by two reviewers (A.v.d.A. and D.A-). Any disagreements were discussed and solved between the two reviewers. Following the aforementioned PRISMA extension for scoping reviews, we did not undertake a risk of bias assessment as part of this scoping review [23].

### Data collection and analysis

Articles were imported into NVivo, where we employed a multi-step coding process based on work by Richards and Hemphill [24]. The first reviewer (A.v.d.A.) conducted a preliminary coding of 50% of the data (28 articles) to develop an initial codebook. The resulting initial codebook was pilot tested by two researchers (A.v.d.A. and D.A.) who independently coded three previously uncoded articles, after which it was revised accordingly. The final codebook (available in Additional file 1: Appendix 1) was then applied to the whole dataset by the first reviewer (A.v.d.A.). It is important to note that the codes were not mutually exclusive, so an article could be coded to multiple codes within the same general theme.

The data were analysed using a general inductive approach (GIA), which is an approach to thematic analysis that consists of both deductive and inductive features. While the general themes are derived deductively from the research objectives, more specific themes arise inductively from the data [25]. This type of thematic analysis has been noted to be useful for summarizing key features within a large dataset [26]. The general themes used to inform the deductive part of the GIA coding were inspired by Waterlander et al. who, in their study on group model building (GMB) described four dimensions across which study designs could vary: (1) the method for defining the initial problem, (2) the structuring of the group process, (3) the type of model and (4) the starting point [27]. We developed the following five general themes (presented in Table 1) as a guiding framework for assessing the included studies: (1) problem definition and goal-setting, (2) participant involvement, (3) structure of the mapping process, (4) validation of the systems map, and (5) evaluation of the mapping process.

**Table 1:** Framework for assessing the included studies

Themes	Items
1) Problem definition and goal-setting	Method for defining the initial problem or research question Purpose of the mapping process
2) Participant involvement	Participant recruitment and selection Types of participants involved
3) Structure of the mapping process	Process of building the systems map Identification of leverage points
4) Validation of the systems map	Method of validation after the map has been built
5) Evaluation of the mapping process	Timing of evaluation Method of evaluation Benefits and limitations arising through evaluation

## Results

**Fig. 1:**
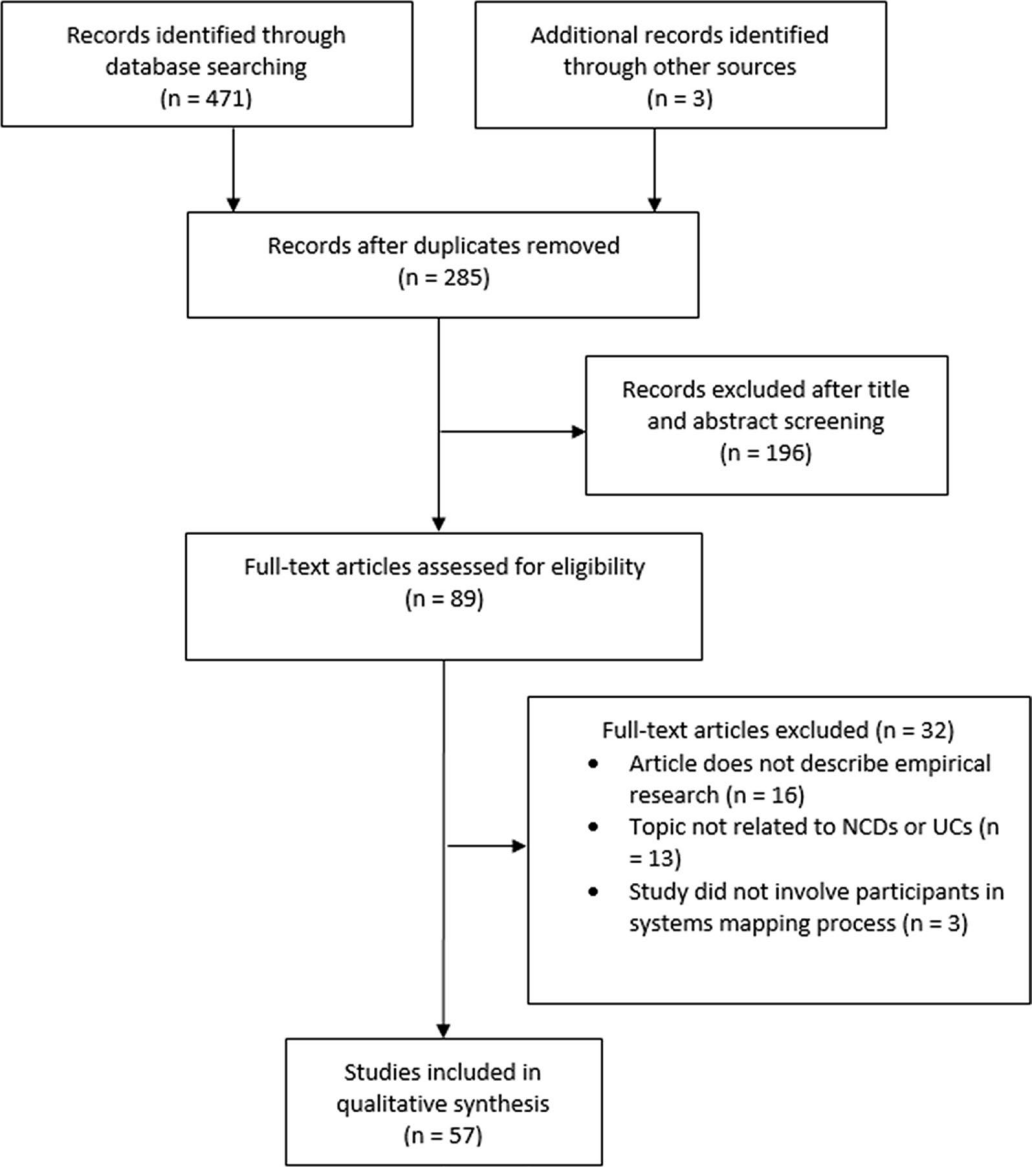
PRISMA flow diagram of included articles

Figure 1 shows that 285 references were identified for screening and 57 met the inclusion criteria; 56% (*n* = 32) of the included studies were published in or after 2020. Table 2 summarizes the characteristics of the included studies. The United States (*n* = 17) and Australia (*n* = 10) were the most common study locations. The most common study topics were obesity, physical activity, mental health, alcohol and NCDs in general. The total number of participants involved in the development of the systems maps within the included studies ranged from 6 to 590.

**Table 2:** Characteristics of the included studies

Authors and year	Journal	Funding source	Study location	Study topic	Total number of participants*	Delivery mode
Allender et al. (2015) [28]	PLoS ONE	National Health and Medical Research Council (NHMRC)	Australia	Childhood obesity	12	In-person
Ansah et al. (2019) [29]	BMC Health Services Research	University	Cambodia	NCDs	25	In-person
Baker et al. (2019) [30]	Obesity Reviews	University	Global	Nutrition	14	In-person
Beks et al. (2022)[31]	Rural and Remote Health	Not reported	Australia	NCDs (chronic disease)	Not reported (NR)	In-person
Bellew et al. (2020) [32]	Journal of Physical Activity and Health	The Medical Research Future Fund (MRFF)	Australia	Physical activity	NR	Not reported (NR)
BeLue et al. (2012) [33]	Health Education and Behavior	No additional funding obtained	United States	Alcohol	NR	In-person
Boelsen-Robinson et al. (2021) [34]	Food Policy	Government and university	Australia	Nutrition	26**	Not applicable***
Brennan et al. (2015) [35]	Journal of Public Health Management and Practice	Robert Wood Johnson Foundation	United States and Puerto Rico	Childhood obesity	590 (avg. 12)	In-person
Calancie et al. (2022) [36]	Journal of Public Health Management and Practice	NHMRC	United States	Childhood obesity	16	In-person
Calancie et al. (2022) [37]	Preventing Chronic Disease	JPB Foundation	United States	Childhood obesity	12	In-person
Cavana et al. (2006) [38]	System Dynamics Review	Not reported	New Zealand	Tobacco	12	In-person
Cavill et al. (2020) [39]	Journal of Public Health Research	Sport England	United Kingdom	Physical activity	12	NR
Chavez-Ugalde et al. (2022) [40]	BMC Medical Research Methodology	National Institute for Health and Care Research (NIHR)	United Kingdom	Nutrition	11	Online
Clarke et al. (2020) [41]	Social Science and Medicine	Australian Research council and Heart Foundation	Australia	Obesity	11**	Not applicable
Clarke et al. (2021) [42]	PLoS ONE	Heart Foundation, NHMRC	Australia	Obesity	57**	Not applicable
Deutsch (2021) [43]	Systems Research and Behavioral Science	National Institutes of Health	Northern Plains Indigenous People	Alcohol and violence	29 (5/20/4)	In-person
Freebairn et al. (2019) [44]	PLoS ONE	NHMRC	Australia	NCDs (Diabetes)	11	NR
Friel et al. (2017) [45]	PLoS ONE	NHMRC	Australia	Nutrition	15	In-person
Gerritsen et al. (2019) [46]	PLoS ONE	Government and university funding	New Zealand	Nutrition	17	In-person
Gillen et al. (2014) [47]	Health Education and Behavior	National Center for Research Resources	United States	NCDs (asthma)	6	In-person
Guariguata et al. (2020) [48]	Nutrients	International Development Research Centre of Canada	The Caribbean	Nutrition	41 (avg 14)	NR
Guariguata et al. (2021) [49]	Policy and Practice	Foreign, Commonwealth and Development Office, MRC, Wellcome Trust, Economic and Social Research Council	The Caribbean	Physical activity	12	In-person
Heke et al. (2019) [50]	AlterNative	Johns Hopkins Global Obesity Prevention Centre	New Zealand	Obesity	15 (8/7)	In-person
Hennessy et al. (2020) [51]	Health Education and Behavior	National Institutes of Health	United States	Obesity	NR	In-person
Hosseinichimeh et al. (2022) [52]	Social Science and Medicine	National Institutes of Health	United States	Alcohol	NR	Hybrid
Hussey et al. (2021) [53]	BMC Health Services Research	No additional funding obtained	Canada	NCDs (COPD and HF)	NR	NR
Idriss et al. (2020) [54]	BMJ Global Health	NIHR	Sierra Leone	NCDs	116 (avg 23)	In-person
Jessiman et al. (2021) [55]	PLoS ONE	NIHR	United Kingdom	NCDs (child health inequalities)	73 (41/32)	Hybrid
Keane et al. (2015) [56]	Journal of Public Health Management and Practice	Robert Wood Johnson Foundation	United States	Childhood obesity	12	NR
Langellier et al. (2019) [57]	Health and Place	Wellcome Trust	10 Latin American countries	NCDs (urban health) and Physical activity	62 (between 16 and 24)	In-person
Matson et al. (2021) [58]	American Journal of Community Psychology	Bloomberg American Health Initiative	United States	Alcohol	30 (9/13/8)	NR
Mills et al. (2021) [59]	Tobacco Control	National Institutes of Health, FDA, CDC, HRSA	United States	Tobacco	19**	Not applicable
Moreland (2015) [60]	Journal of Public Health Management and Practice	Robert Wood Johnson Foundation	United States	Childhood obesity	10	In-person
Mui et al. (2019) [61]	PLoS ONE	Johns Hopkins Urban Health Institute, CDC	United States	Nutrition	18	NR
Nelson et al. (2015) [62]	Journal of Public Health Management and Practice	Robert Wood Johnson Foundation	United States	Childhood obesity	26	NR
Noubani et al. (2021) [63]	International Journal of Mental Health Systems	NIHR	Lebanon	NCDs (mental health)	21 (9/12)	In-person
Odland et al. (2020) [64]	World Journal of Surgery	NIHR, University, SRMRC	Rwanda	NCDs (injury)	34	In-person
Owen et al. (2018) [65]	PLoS ONE	National Institutes of Health USA, NHMRC	Australia	Childhood obesity	16**	Not applicable
Parmar et al. (2021) [66]	BMJ Open	Research for Health in Humanitarian Crises	Jordan	NCDs	20**	In-person
Poon et al. (2022) [67]	Health and Place	Social Sciences and Humanities Research Council	Canada	NCDs (child mental health)	31 (avg. 14)	In-person
Ramsey et al. (2019) [68]	BMJ Open	National Institute of Drug Abuse (NIDA)	United States	Tobacco	50 (avg. 12)	NR
Riley et al. (2021) [69]	Systemic Practice and Action Research	NHMRC	Australia	NCD (chronic disease)	NR	NR
Rwashana et al. (2014) [70]	Health Research Policy and Systems	International Development Research Centre, Canada	Uganda	NCDs (neonatal mortality)	327**	NR
Savona et al. (2021) [71]	The European Journal of Public Health	European Union Horizon 2020 research and innovation programme for Sustainable Food Security	5 European countries	Obesity	257 (avg. 13)	NR
Sharma et al. (2020) [72]	BMC Public Health	University	Nepal	Tobacco and alcohol	NR	NR
Skouteris et al. (2015) [73]	Australian and New Zealand Journal of Obstetrics and Gynaecology	NR	NR	Obesity	NR	In-person
Stansfield et al. (2021) [74]	Journal of Public Mental Health	The Health Foundation	United Kingdom	NCDs (mental health)	40	In-person
Suriyawongpaisal et al. (2021) [75]	Public Health in Practice	Thai Health Promotion Foundation	Thailand	Alcohol	11	NR
Swierad et al. (2020) [76]	Journal of Obesity	CUNY SPH	United States	Childhood obesity	16	NR
Thomas et al. (2015) [77]	Journal of Public Health Management and Practice	Robert Wood Johnson Foundation	United States	Childhood obesity	21	In-person
Trani et al. (2016) [78]	Conflict and Health	Not reported	Afghanistan	NCDs (mental health)	14 (6/4/4)	In-person
Uleman et al. (2021) [79]	GeroScience	American Alzheimer’s Association	Not reported	NCDs (alzheimer’s disease)	15	NR
Urwannachotima et al. (2019) [80]	Systems Research and Behavioral Science	Thailand Research Fund (TRF)	Thailand	Sugar-sweetened beverages	10	In-person
Waqa et al. (2017) [81]	Health Research Policy and Systems	NHMRC Australia	Fiji	Nutrition	18 (9/9)	In-person
Williams et al. (2018) [82]	Journal of Health Disparities Research and Practice	TREC Center, University, National Cancer Institute	United States	NCDs (cancer)	34	In-person
Witter et al. (2020) [83]	Conflict and Health	NIHR	Sierra Leone	NCDs	80 (avg 27)	In-person
Zablith et al. (2021) [84]	Conflict and Health	NIHR	Lebanon	NCDs	37 (avg 12)	In-person

*When multiple sessions were held, the number of participants per session is represented in brackets

**Participants were separate interviewees, not in a group

***Where the delivery mode is indicated as ‘not applicable’, the method of creating the systems map did not include group workshops with participants

### Problem definition and goal-setting

In their rationale for taking a systems approach, most authors referred to the complexity of the problem (*n* = 33)[28–30, 32–40, 42, 45, 47–49, 51–53, 55, 57, 58, 61, 65, 69–71, 76, 78, 79] alongside a need for interventions or policies that are community-based (*n* = 10) [31, 43, 46, 50, 54, 58, 63, 67, 68, 76], cross-sectoral (*n* = 10) [29, 36, 42, 55, 66, 73, 74, 79–81], focused on upstream solutions (*n* = 13) [32, 35, 39, 45, 47, 50, 59, 67, 70–72, 75, 77], or a combination of any of these. The majority of included studies (*n* = 30) used systems mapping to gain an in-depth understanding of this system [32, 33, 37, 39, 41, 42, 45–48, 52, 53, 55, 57–59, 61–64, 69–72, 75, 79–81, 83] and approximately half of those (*n* = 14) also sought to identify leverage points [31, 33, 34, 40, 45, 46, 48, 57, 61, 68, 70, 71, 74, 81]. Other studies used participatory systems mapping to evaluate a project, intervention or policy (*n* = 12) [34, 35, 39, 51, 56, 59, 60, 62, 65, 66, 77, 81], to inform a new intervention (*n* = 7) [28, 36, 37, 40, 44, 73, 78], or to validate existing frameworks (*n* = 4) [30, 48, 54, 64]. Four studies specifically conducted a participatory systems mapping process to develop a quantitative model, usually with the purpose of simulating the impact of a certain policy or intervention [29, 38, 44, 80]. The starting point for the systems mapping exercise was often based on decisions made by the core research team or preliminary literature reviews. In eight studies, this was instead based on discussions with participants [31, 46, 48, 69, 73, 74, 80, 83], and in eight studies defining the goal was part of the systems mapping exercise itself [33, 36, 38, 44, 50, 57, 67, 81].

### Participant involvement

Most studies recruited participants purposively, often based on their profession or experiences (*n* = 32) [28, 29, 31, 38, 40–42, 45, 46, 49, 54, 56–58, 61, 63, 64, 66–68, 70–72, 74–76, 78–84] or because of their involvement in a certain project (*n* = 14) [33–37, 39, 43, 50, 51, 58, 60, 69, 70, 77]. Authors often used local non-governmental organizations (NGOs), community organizations or previous interviewees to recruit participants. Eleven studies did not specify how they recruited participants [30, 32, 44, 47, 48, 50, 52, 53, 62, 65, 73]. The participant groups that were invited to participate most often were policy makers (*n* = 30) [29, 32, 34, 35, 38–42, 44, 45, 48, 49, 54, 55, 57, 58, 60, 61, 64, 66, 67, 70, 72–75, 80, 81, 83] and professionals (*n* = 31) [28–30, 34, 36, 37, 39, 40, 44, 46, 49, 50, 53–56, 63–68, 70, 72, 75–78, 82–84], with the latter including mainly healthcare professionals or education professionals. Other commonly included groups of participants were community members (*n* = 28) [28, 31, 35, 40, 43, 46, 48, 50, 53–56, 58–62, 66, 68–73, 76, 77, 82, 84], local NGOs (*n* = 20) [29, 32, 35, 39, 41, 42, 45, 48–50, 55–58, 61, 62, 64, 66, 72, 75, 77] and academics (*n* = 18) [29, 35, 41, 42, 45, 49, 51, 56–59, 62, 64, 67, 73, 77, 79, 80].

### Structure of the mapping process

There were significant differences in the participatory systems mapping processes in the included studies. In fact, almost none used the same process. As such, it proved impossible to capture all procedural nuances and instead we have categorized the processes under broad headings. These should be read with the understanding that there are procedural differences even between studies that fall under the same heading.

In approximately half of included studies, participants built the systems map during the process, using a variety of activities or ‘scripts’ (*n* = 27) [29, 31, 33, 37, 38, 40, 43, 44, 47, 56–58, 60, 62, 63, 66, 67, 73, 74, 77–84]. These scripts were usually taken and amended from Scriptapedia, a free online repository [85]. The most commonly used scripts were variable elicitation, creating graphs over time, prioritizing variables and creating causal feedback loops. In 13 studies participants built the map which researchers later amended or supplemented [28, 35, 45, 46, 48, 49, 54, 55, 61, 63, 68, 71, 76]. In three studies, participants provided variables during the participatory workshop, but did not build the systems map, which researchers built later [36, 50, 52]. When researchers built the systems map prior to the participatory mapping exercise, they did so based on existing literature and/or document review (*n* = 3) [30, 39, 59] based on participant input, for example through interviews (*n* = 4) [34, 65, 66, 72], or based on both literature review and participant input (*n* = 11) [32, 39, 41, 42, 51, 53, 55, 64, 69, 70, 75].

Of the 28 studies that included the identification of leverage points, this was mostly done by participants (*n* = 23) [31, 37, 39, 40, 44, 46, 48, 49, 57, 58, 60, 61, 63, 66, 68, 69, 73, 74, 76, 78, 81, 82, 84]. Of these, nine studies asked participants to not only identify, but also prioritize leverage points [37, 44, 46, 48, 57, 61, 68, 74, 84]. In four studies the researchers identified leverage points, and in two studies it was unclear who identified leverage points [32, 33].

### Validation of the systems map

In 34 studies, the final systems map was presented to participants for feedback in order to validate the map [28–30, 36, 39, 40, 44–46, 48, 50–53, 55, 57–59, 61, 64–70, 72, 73, 75–77, 79, 80, 84]. Other methods used for validation were to map the systems map onto an existing (theoretical) framework (*n* = 7) [41, 42, 49, 54, 63, 78, 83], or to triangulate the map with other data, mostly interviews and/or scientific literature (*n* = 11) [34, 35, 40, 49, 52, 63, 64, 68, 74, 78, 79]. In a number of studies multiple maps were created, which were consolidated or compared against one another [43, 55, 65]. The integration of different maps was usually followed by another method of validation, such as follow-up with participants by email or in a workshop setting. Ten studies did not state whether they validated the systems map after completion [31–33, 37, 47, 56, 60, 62, 69, 71, 81].

### Evaluation of the mapping process

The majority of the included studies (*n* = 48) did not evaluate the participatory mapping process. Of those who did, five conducted an evaluation after the process [33, 39, 48, 50, 57], and four did this both during and after the process [36, 37, 40, 48]. Semi-structured interviews were the most common method of evaluation (*n* = 6) [33, 36, 37, 39, 48, 57]. Of these, three studies supplemented interview findings with a questionnaire [36, 37, 48]. One study used group discussions [50] and one study used both surveys and group discussions as a method of evaluation [40]. Table 3 sets out the benefits and limitations of participatory systems mapping that were identified through these evaluations. Benefits mostly related to changes in participants’ perspective on the issue, increased knowledge on the topic and building connections between participants. Some participants discussed limitations of the method, including concerns that the systems map might not lead to action, particularly when there is a lack of buy-in from powerful actors who might effectively translate the results to policy action.

**Table 3:** Benefits and limitations of participatory systems mapping identified in evaluations

Benefits	Limitations
A better understanding of the complexity of the issue	Does not explicitly lead to action
Exposure to other perspectives on the issue	Difficult to implement results in real life
Change in their own perspective on the issue	Incomplete participant representation
Creates space for cross-sectoral dialogue and work	Lack of buy-in from powerful actors
Enhanced awareness of others working in the same area	Method takes large time commitment
Increased knowledge on the topic	Insufficient time for discussion
Method accessible and stimulating	Method complex and challenging to understand
Builds connections between participants	
Increased trust between participants	
Increased trust between participants and researchers	
Creates ownership over the outcomes of the process	

## Discussion

Of the 57 studies included in this review, 32 were published in or after 2020, indicating an increased academic interest in participatory system mapping methods in the context of NCDs and associated risk factors. Most researchers used a systems approach to gain a more ‘upstream’ understanding of a complex problem, such as obesity of physical inactivity, or to gain a community’s perspective on an issue. The role of participants within the systems mapping process varied widely. Some studies involved participants in all stages of the systems mapping process from goal-setting through to building the map and identifying leverage points, while in some other studies participant involvement was limited to providing one-off input on a pre-made systems map. In 17 studies goal-setting or problem definition was based on discussions with participants either separate from or as part of the participatory process. The research question, framing and boundaries of the systems map can have a significant impact on the systems mapping process. Existing guidelines on how to conduct participatory systems mapping processes provide a general structure of the process [19, 86, 87], while appreciating that there will be variety in how these processes are conducted, depending on the needs and purpose of the project [17, 87, 88]. A different research aim or focus within system can lead to different specific questions being asked during the mapping process, can require different participants to be involved, and result in different outcomes [17, 89]. Ideally, formulating the project aim and defining system boundaries is done together with stakeholders to ensure the relevance of the map’s focus and increase ownership and commitment to the process by participants [90].

Recent methodological guidance on participatory systems mapping emphasizes the importance of including a multidisciplinary, diverse and representative group of participants to create a comprehensive and inclusive systems map [17]. The participant groups most often involved in the included studies were policy makers and professionals. There are certain benefits to including these traditionally powerful actors, who may be key for translating the systems map into action [46]. However, systems thinking in itself does not necessarily challenge traditional worldviews or ‘blind spots’ [91]. Various authors have emphasized the importance of including a diversity of participants, who may view the problem through a different lens, challenge established narratives or norms, uncover and discuss conflicting perspectives or identify non-conventional approaches to a problem [17, 18, 92]. Engagement with the complex system it seeks to map is an integral part of a mapping approach, which may be particularly important when it includes marginalized or vulnerable communities.

The flexibility of the systems mapping process allows researchers to use the method in combination with non-Western methods of engagement and knowledge-sharing. For example, Beks et al. incorporated the Aboriginal and Torres Strait Islander ‘yarning’ method in their systems mapping process [31]. As Heke et al. argue, a key strength of participatory systems mapping is that it can potentially provide a ‘bridge’ between traditional and non-traditional, or indigenous, knowledge bases [50]. While systems mapping has been found useful in effectively engaging a variety of participants and communities, this does require researchers to be especially cognisant of existing power imbalances or potential misunderstanding about motives [50, 93]. As one of the included studies mentioned, inviting community members or people with lived experience, who may traditionally be marginalized, into a group of experts may invoke power imbalances and inhibit community members’ participation [43]. Nevertheless, among most of the studies that included both traditionally powerful actors and community members, this consideration was not explicitly addressed. Future research using participatory systems mapping would benefit from at least some acknowledgement of these potential power imbalances, which includes those between researchers and participants. Ideally, this would extend to some exploration of strategies to mitigate these imbalances throughout the systems mapping process. Existing research on promoting inclusiveness and equality in participatory research processes highlights the benefits of involving participants throughout all stages of the process, being transparent about the aim of the research, being aware and transparent about relationship asymmetries, using accessible language or picture-based story-telling techniques, having an experienced facilitator to manage group dynamics, and returning the results of the process to participants for them to provide feedback outside the group setting [17, 90, 94–96].

The majority of studies included in this review conducted their systems mapping workshops in-person. However, the shift to online working and learning during and after the COVID-19 pandemic has shown that conducting online workshops is both possible and at times beneficial. Some of the studies included in this review reported conducting their systems mapping workshops online or both in-person and online [40, 52, 55]. Facilitating online systems mapping workshops comes with a range of opportunities and challenges. One challenge is that it requires participants to have access to a stable internet connection to be able to participate. This may be a barrier to access, particularly for those from rural areas or disadvantaged communities [97, 98]. Moreover, researchers have noted that online workshops provide limited interaction; this may favour participants who are more fluent or confident speakers and may lead to fatigue both on the part of participants and facilitators [17, 99]. Nevertheless, conducting online participatory systems mapping workshops may increase participation, as participants do not have to travel to a central location, and adaptations can be made to better facilitate the online nature of the workshop, such as by having more, shorter mapping sessions [17].

While nearly all studies engaged with participants after completion of the systems map in some way to validate the systems map, only a minority of studies reported having undertaken participant evaluation of the process. Of these, the focus of evaluation differed from asking participants about their own experiences during the mapping exercise to asking participants how useful the exercise had been for their work and whether their perspective on a topic had shifted as a result of the workshop [36, 40, 50]. From the participant evaluations that were reported, many of the reported benefits of participatory systems mapping related to individual or group learning. This is in line with findings by Scott et al. who reported on the effectiveness of the method in achieving group decisions, and adding to individual and group learning, noting changes in participant behaviour and participant learning [22]. On the other hand, the limitations described by participants of the included studies highlight the difficulties of translating systems mapping results into policy action: although participatory systems mapping processes may generate useful knowledge in the form of a systems map or potential leverage points, this learning may not lead to changes in policy or practice. Participants reflected on the method as part of a wider policy process and mentioned that the outcomes of the process may be difficult to implement in real life.

In one study, these limitations were linked to a lack of buy-in from powerful actors, such as community leaders or policy makers who have the authority to enable action [93]. Participants of another study noted that the mapping exercise would be most useful to inform the planning of a new policy or programme, noting difficulties with modifying policy or programme components to address the issues identified in the mapping process [33]. Participants of several studies reflected that while the complexity inherent to systems approaches may at times be difficult to translate into concrete policy action, the process and its outcomes, often leading to novel insights into the complexity of the problem, a sense of ownership by the participants, building relationships between participants and changes in perspectives, was found to be highly valuable in the policy process [37, 39, 40, 92]. There is a large literature base on the implementation of participatory processes into policy making, which reflects the issues noted in some of the included studies and emphasizes the importance of understanding the context within which the process takes place, identifying current gaps or policy asks and including the right stakeholders in the process to enable policy action [7, 92, 100–103]. A recent study on the experiences of policy makers who joined in a partnership taking a systems approach to NCDs in Australia highlighted that policy makers agreed that for systems thinking to be of most added value to their policy work, the focus should not be on documenting a complex system, but rather on identifying ways to intervene in this system [7]. The benefits of identifying leverage points as part of the systems mapping process was highlighted by authors and participants in several studies [35, 40, 47, 48, 67, 68, 77–79]. A common argument was that identifying leverage points, or explicitly designing actions, during the systems mapping process facilitates action as it can lead to concrete recommendations. This may increase motivation to act by those who were involved in the formulation of leverage points themselves [40, 78, 93].

While the flexibility of participatory systems mapping method is one of its key strengths, there is scope for further exploration of the usefulness of specific scripts or activities for specific purposes or audiences. It is interesting to note some contradictory feedback in the evaluation of the included studies. For example, the mapping process has been described as both ‘too time-consuming’ and as not providing enough time for participants to fully engage. The method has also been perceived by different participants as both ‘too complex’ and ‘accessible and stimulating’. The limited number of studies that reported on evaluations precludes us from drawing any meaningful conclusions as to how the use of different methods relates to such opposing feedback, which may be a useful area for future research. By reviewing the use of participatory systems mapping as a research method in the context of NCDs and their risk factors, we have found a wide variety of methodological approaches. The flexibility of the method is one of its strengths, as it allows for adaptation to the research context. However, based on our findings we propose that future research using participatory systems mapping approaches may benefit from careful consideration of the key issues highlighted throughout this paper. This includes for researchers to ensure that participatory systems mapping is an appropriate method for the context, to include a diverse range of stakeholders throughout the process, for the facilitator to remain conscious of power relations both between participants and participants and the research team, and ensure that everyone has an equal chance to contribute.

Our study carries several limitations. As with any academic literature review, there is the risk that relevant studies have been missed. In the current context this risk is especially pronounced as participatory systems mapping methods are often used in a practical context, which may not always be published in academic journals. This risk was increased by having a single reviewer in the title and abstract stage, although the full-text screening was conducted by two reviewers independently, which helped mitigate this risk.

## Conclusion

The current study found an increase in the use of participatory systems mapping, for a variety of purposes and including a wide variety of participants, both in number and type of stakeholders. While a lack of formal evaluation made it difficult to draw conclusions on participant experiences with the wide range of approaches used within participatory systems mapping, most benefits mentioned by participants related to individual or group learning, while limitations related to the position of participatory systems mapping as part of the wider policy process. We summarized published data on the use of participatory systems mapping in the context of NCDs and UCs. In doing so, this review engaged with a rapidly growing interest in taking a systems approach to address the complexity of these issues, as evidenced by the recent WHO publication on using systems mapping to inform NCD prevention policy [11].

Various authors noted the benefits of including community members or otherwise marginalized groups to gain new insights into the system, while also noting the need to include traditionally powerful actors such as policy makers or professionals to enhance the potential for the mapping process to result in action [31, 43, 50]. This apparent trade-off leads to important questions on representation and power and how they might impact the systems mapping process. Some authors noted a gap between the emphasis on complexity in systems mapping outcomes and the practical realities of policy making, which may interfere with the ability of systems mapping to drive policy [46]. More needs to be known in terms of to what extent, for which purposes and in which contexts participatory systems mapping methods can lead to meaningful insights, understanding or change. With increasing interest in participatory systems mapping methods as a tool for tackling complex problems, there is a need for better understanding of what is required for the method to be an effective, representative and fair process that can make significant contributions to meaningful systems change.

## Implications for policy and practice

- There is increasing interest in the use of participatory systems mapping methods as a useful tool in the context of NCDs and related risk factors, as it recognizes the complexity of the problem and often invites a diversity of perspectives on these issues.
- Participatory systems mapping hold potential value for stakeholder engagement, as the included studies included a wide variety of participants, both in numbers and in type of stakeholders. However, very few studies discussed the impact that participant composition had or might have had on the process.
- While a general lack of formal evaluation makes it difficult to draw conclusions, reported benefits by participants generally relate to participant or group learning and building of connections, with participants noting the difficulty of translating systems mapping results into action as one of the limitations of the approach.

## Supplementary Information

**Additional file 1:** Codebook of themes in scoping review.

## Data Availability

All data analysed during the current study are included in this published article.

## Abbreviations

NCD: Non-communicable diseases
WHO: World Health Organization
CLD: Causal loop diagrams
CBSD: Community-based system dynamics
GMB: Group model building
GIA: General inductive approach
NGO: Non-governmental organization
UC: Unhealthy commodity
