# Supplementary material for: The use of participatory systems mapping as a research method in the context of non-communicable diseases and risk factors: a scoping review
Source: Health Res Policy Syst. 2023 Jul 6;21:69. doi: 10.1186/s12961-023-01020-7 (PMC10327378; doi:10.1186/s12961-023-01020-7)
Supplement: Supplementary file 1 — Additional file 1: Codebook of themes in scoping review. [file 12961_2023_1020_MOESM1_ESM.docx]

# Codebook Scoping Review Participatory systems mapping, V1

| **Themes** | **Subthemes** | **Definitions** | **Examples from Transcript** |
| --- | --- | --- | --- |
| **A) Problem definition and goal-setting** | | | |
| **A1**  Method for defining the initial problem or research question | **A1.1**  Based on theoretical framework | The goal/topic of the participatory mapping process is based on a theory or a theoretical framework | “To inform the development of the model on political commitment, we drew on several complementary theories applied in nutrition policy research.” #153 |
|  | **A1.2**  Based on preliminary literature review | The goal/topic of the participatory mapping process is based on a literature review. This includes academic literature or grey literature | “Development of research questions and design of the study were informed by findings from a scoping review that was done earlier” #191 |
|  | **A1.3**  Based on core research team | The research team defines the goal and topic of the mapping process | “A core modelling team […] defined the research aim and objectives and developed the study protocol.” #27 |
|  | **A1.4**  Based on discussions with local community | The goal and objectives of the participatory mapping process are based on preliminary discussions with stakeholders, prior to the mapping process. | “the genesis of the systematic scoping review question arose in early discussions around geographical allocation of funding with local Aboriginal stakeholders” #38  “A total of 70 semi-structured face-to-face interviews were conducted with stakeholders […] [to] inform the framing and planning of the workshops.” #160 |
|  | **A1.5**  Goal-setting as part of the participatory mapping process | Setting the goal, topic and/or objectives of the participatory systems map is integrated into the mapping process | “This tutorial was followed by a participatory mapping process led by Dr. Booth Sweeney to clarify the goals of the systems mapping exercise” #175 |
|  | **A1.6**  Not mentioned |  |  |
|  | **A1.7**  Other | The goal, topic and/or objectives of the systems map are defined by a different means |  |
| **A2**  Framing | **A2.1**  Focus on complexity | Within the framing of the problem and/or rationale for the systems map and/or employing a participatory systems mapping process, there is a focus on complexity | “There is no doubt that health care systems demonstrate complex system characteristics” #188  “this study aimed to organise, from a complex systems perspective” #137 |
|  | **A2.2**  Focus on need for upstream solutions | Within the framing of the problem and/or rationale for the systems map and/or employing a participatory systems mapping process, there is a focus on the need for upstream solutions (/root causes/ fundamental solutions/social determinants) | “The purpose of this study is to create a CLD […] that encompasses individual,  environmental and  root cause or ‘fundamental’ factors.” #274 |
|  | **A2.3**  Focus on individual behaviour | Within the framing of the problem and/or rationale for the systems map and/or employing a participatory systems mapping process, there is a focus on individual behaviour and its role in the system | “interventions on behavioural change that focuses on tackling NCDs risk factors” #11  “focused on eliciting perceptions on: i) the characteristics of persons suffering from chronic conditions, ii) the factors leading to the onset of such conditions and iii) the help and health-seeking journey of people living with NCD” #462 |
|  | **A2.4**  Focus on need for cross-sectoral action | Within the framing of the problem and/or rationale for the systems map and/or employing a participatory systems mapping process, there is a focus on the need for multi-disciplinary or cross-sectoral action | “will require systemic approaches that transcend disciplines and sectors.” #160 |
|  | **A2.5**  Focus on need to understand community perspective |  | “However, little is known about the experiences and attitudes of currently active health care providers” |
|  | **A2.6**  Other |  |  |
| **A3**  Purpose of the participatory mapping process | **A3.1**  Inform or develop intervention or policy | The purpose of the mapping process is to inform or develop a new intervention or policy | “Using participatory systems diagramming techniques before intervention planning” #153 |
|  | **A3.2**  Evaluate intervention or policy | The purpose of the mapping process is to understand or evaluate the effects of an intervention or policy | “to anticipate the intended and unintended effects of tobacco control policies” #274  “participants wanted to understand the root causes of childhood obesity to evaluate where MCOPP is making an impact” #291 |
|  | **A3.3**  Compare an existing framework with reality | The purpose of the mapping process is to ‘test’ the map against a theory or theoretical framework | “to explore how they fit in a previously described framework” #191 |
|  | **A3.4**  Develop quantitative model | The purpose of the mapping exercise is to develop a quantitative model. Note: This only applies when authors specifically plan or aim to develop a quantitative model. | “a first step for developing a quantitative simulation model” #11  “we plan to simulate the model and use the model to ask “what-if” questions” #183  “using a participatory process to transform qualitative conceptual maps of diabetes in pregnancy into a quantified dynamic simulation model.” #134 |
|  | **A3.5**  Gain in-depth understanding of a system | Through the mapping exercise, researchers/organisers aim to gain a more in-depth understanding of a system or participants’/patients’ experiences with a system | “aim to gain an in-depth understanding of NCD management in Cambodia” #11  “describe delays and identify and prioritise barriers to accessing quality injury care in Rwanda and to visually represent the complex inter-relationships between them.” #302 |
|  | **A3.6**  Identify leverage points within a system | The organisers aim to identify leverage points within a system. Note: This only applies when identifying leverage points is explicitly part of the current mapping process | “with a view to identify opportunities for strengthening mental health and psychosocial support service (MHPSS) implementation.” #300 |
|  | **A3.7**  Gain insight into participants’ perspectives of a specific issue or policy | The purpose of the mapping exercise is to gain insight into participants’ perspectives and/or experiences on a specific issue and/or policy or intervention | “This study aims to understand diverse stakeholders' perspectives on the alcohol zoning policy […] and to gain insights into their understanding of the mechanisms and potential ripple effects” #260 |
|  | **A3.8**  Validate a pre-made model | The participatory aspect of the systems mapping process is meant to validate a pre-made model. Note: this code applies when participants’ input is only used to validate a pre-made model. | “a workshop with experts to strengthen the face validity of the models” #27 |
|  | **A3.9**  Other |  |  |
| **B) Participant involvement** | | | |
| **B1**  Participant recruitment and selection | **B1.1**  Based on key informant interviews | Participants are selected based on interviews and/or discussions with other stakeholders. This can be done through discussions with a stakeholder group / NGO or through snowball sampling. | “The convenience sample was recruited using snowball sampling, whereby stakeholders interviewed were asked to name others” #274 |
|  | **B1.2**  Based on project participants | Participants are included based on their involvement within a specific project/intervention/policy | “The experts were linked by their involvement in The Australian Prevention Partnership Centre” #137  “HKHCuba partners were invited by the HKHCuba project staff to participate in a group model building (GMB) session” #210 |
|  | **B1.3**  Purposive sampling | Participants are purposively selected to represent a specific experience or role | “Participants were sampled purposively, selecting specific experiences and/or roles and functions performed within the local community” #191  “Participants were also targeted for recruitment based on their expertise in either food or transport systems” #233 |
|  | **B1.4**  Other | Participants are recruited based on different criteria, not mentioned above |  |
|  | **B1.5**  Not specified | The basis for participant recruitment is not specified in the article |  |
| **B2**  Types of participants involved | **B2.1**  Academics | | |
|  | **B2.2**  Policy-makers | | |
|  | **B2.3**  Topic experts | | |
|  | **B2.4**  Professionals | | |
|  | **B2.5**  Community members | | |
|  | **B2.6**  Community leaders | | |
|  | **B2.7**  Local NGO | | |
|  | **B2.8**  Global NGO/ international organization | | |
|  | **B2.9**  Private sector | | |
|  | **B2.10**  Small or medium enterprise | | |
|  | **B2.11**  Computer scientists | | |
|  | **B2.12**  Other | | |
| **C) Structure of the mapping process** | | | |
| **C1**  Used script | **C1.1**  Developed by experts or researchers | The researchers develop the scripts, without relating specifically what these scripts are based on. | “The Transtria modeling team provided the outline and goals of the GMB session” #210  “We developed a new script (e.g., a facilitation guide outlining how to conduct the GMB activities)” #115 |
|  | **C1.2**  Taken from repository (and amended) | The researchers have taken scripts from a script repository (e.g. scriptapedia). They might have amended the script but do not specifically state based on what they have amended the script. If they do specifically state what the amended script is based on, use one of the codes above. | “Scripts were elaborated and refined by the research group and the majority draw on publicly available scripts (Scriptopaedia)” #191  “adapted from Scriptapedia to fit the SUU5 context in consultation with the process coaches.” #82 |
|  | **C1.3**  Based on literature of other researchers’ experience | The researchers develop of modify scripts based on literature on previous participatory mapping exercised by different authors, where the authors are specifically mentioned. | “We were guided by the principles and strategies outlined by Vennix and Luna-Reyes et al. in  structuring the workshops” #160 |
|  | **C1.4**  Based on key informant interviews | The researchers develop or modify scripts based on interviews they have held during the current study. | “The GMB sessions included a series of exercises, each based on a specific script (Additional file 1), which were refined based on emerging insights from interviews.” #462 |
|  | **C1.5**  Based on current study context | The researchers develop or modify scripts based on the current context or workshops that have been held during the current study. | “The project employed an iterative approach whereby the structure of each workshop was slightly altered based on context and lessons learned from preceding workshops” #233  “The overall format of the GMB sessions were uniquely tailored to each stakeholder group” #260 |
|  | **C1.6**  Not mentioned | The authors do not make it clear what scripts are based on, for example by merely stating that ‘multiple scripts were used’. | “multiple scripts were used to extract key variables and potential policies, and to conceptualize the feedback processes” #183 |
|  | **C1.7**  Other |  |  |
| **C2**  Process | **C2.1**  Participants build systems map | Participants build the systems map themselves, during the workshop. | “Key members were invited to participate in a half-day GMB workshop to create behavior-over-time graphs and a causal loop diagram” #283 |
|  | **C2.2**  Participants build map which researchers amend afterwards | Participants build the systems map, but researchers edit/refine/amend this map later. | “Diagrams were iteratively refined using the notes taken during sessions and consolidated into one comprehensive CLD” #300 |
|  | **C2.3**  Researchers build map based on participant input during workshop | Researchers build the systems map based on input from participants during the workshop (e.g. variables, feedback loops, rounds of feedback) | “specific focus groups were conducted to elicit a first round of feedback on the base model” #188 |
|  | **C2.4**  Researchers build map based on participant input and/or interviews prior to workshop | Researchers build the systems map based on participant input (e.g. through interviews or discussions) that was obtained prior to the workshop | “conducted 1v1 interviews with various members of the SUS systems map Team to further develop the map” #175 |
|  | **C2.5**  Researchers build map based on literature review | Researchers build the systems map based on analysis of existing literature or documents | “CLD development was informed by a literature review and the theoretical frameworks described above” #274 |
|  | **C2.6**  Researchers build map based on both literature review and participant input | Researchers build the systems map based on an analysis of the literature as well as input obtained from participants either before or during the workshop | “Data collection involved semi-structured interviews with stake-holders involved in relevant policy processes, and analysis of relevant documents pertaining to the policy processes” #100 |
| **C3**  Leverage points | **C3.1**  Participants identify leverage points | Participants identify leverage points within the systems map |  |
|  | **C3.2**  Participants identify and prioritize leverage points | Participants identify leverage points within the systems map and prioritize or rank these |  |
|  | **C3.3**  Researchers identify leverage points | Researchers identify leverage points within the systems map |  |
|  | **C3.4**  Researchers identify leverage points, participants prioritize leverage points | Researchers identify leverage points within the systems map, which participants prioritize or rank |  |
|  | **C3.5**  Other | Leverage points are identified through other means |  |
|  | **C3.6**  No leverage points identified | There are no leverage points identified in the systems map. |  |
| **D) Analysis of the systems map** | | | |
| **D1**  Method of validation after the map has been built | **D1.1**  Participants provide feedback on systems map | Researchers construct or amend the map, after which participants are asked to provide feedback and/or add to the map. | “In groups, participants were asked to critique the collective CLD” #146 |
|  | **D1.2**  Model mapped onto existing (theoretical) framework | The researchers use existing theoretical frameworks / theories to make sense of the systems map data. | “the barriers proposed at the workshop were synthesized into overarching categories by authors based on established health system frameworks” #302  “The patterns also align well with the recommendations of the WHO action plan on physical activity. […] Our summary causal loop diagram extends that framework” #163 |
|  | **D1.3**  Triangulation with other data – various sources | The researchers triangulate the data generated by the participatory mapping workshops with other data to generate the systems map. This additional data may come from various sources (e.g. interviews, literature review, project data) | “The knowledge base underlying the systems map consists of two components: (1) qualitative and quantitative data collected during the intervention phase and (2) qualitative data generated by the SUS systems map team, during group discussions and semistructured interviews” #175 |
|  | **D1.4**  Other |  |  |
|  | **D1.5**  Not mentioned | No further methods are used to validate the systems map |  |
| **E) Evaluation of the mapping process** | | | |
| **E0**  No evaluation has taken place | | | |
| **E1**  Timing of evaluation | **E1.1**  Evaluation during the process | Participants are asked to evaluate the participatory mapping process during the process | “The online survey was fielded at 3 time points” #82 |
|  | **E1.2**  Evaluation after the process | Participants are asked to evaluate the participatory mapping process after the process is concluded |  |
| **E2**  Method of evaluation | **E2.1**  Surveys | Participants are asked to complete surveys/questionnaires as part of the evaluation | “At the end of the workshops, stakeholders were asked to complete an evaluation of the GMB session using a short, voluntary, and anonymous online survey” #160 |
|  | **E2.2**  Semi-structured interviews | Researchers conduct semi-structured interviews with participants as part of the evaluation | “Stakeholders were also interviewed before and after the workshops to provide their perspectives on the utility of this approach” #160 |
|  | **E2.3**  Group discussion | Participants are asked to evaluate the process in a group discussion | “This was explored verbally with the policymakers during the session.” |
|  | **E2.4**  Other | The process is evaluated through other means |  |
| **E3**  Benefits arising through evaluation | **E3.1**  Applicability of systems thinking approach in wider work | | |
|  | **E3.2**  Awareness of others working on similar issues | | |
|  | **E3.3**  Better understanding of the complexity of the issue | | |
|  | **E3.4**  Build new connections between participants | | |
|  | **E3.5**  Capacity-building for participants | | |
|  | **E3.6**  Change in perspective | | |
|  | **E3.7**  Changing priorities in own work | | |
|  | **E3.8**  Create shared language or understanding of the topic | | |
|  | **E3.9**  Create space for cross-sectoral dialogue and work | | |
|  | **E3.10**  Exposure to other perspectives | | |
|  | **E3.11**  Higher engagement with topic | | |
|  | **E3.12**  Increase in knowledge of topic | | |
|  | **E3.13**  Increase in trust | | |
|  | **E3.14**  Method accessible and stimulating | | |
|  | **E3.15**  Participants feel ownership over results of the workshop | | |
|  | **E3.16**  Results of participatory process well received by policy-makers | | |
|  | **E3.17**  Space for conflicting perspectives to emerge and be discussed | | |
|  | **E3.18**  Strengthen existing relationships between participants | | |
|  | **E3.19**  Other | | |
|  | **E3.20**  Benefits not described | | |
| **E4**  Limitations arising through evaluation | **E4.1**  Actions not explicitly following from process | | |
|  | **E4.2**  Difficult to implement results in real life | | |
|  | **E4.3**  Incomplete participant representation | | |
|  | **E4.4**  Lack of buy-in from powerful actors who are not involved in process | | |
|  | **E4.5**  Lack of support from their organization | | |
|  | **E4.6**  Large time commitment | | |
|  | **E4.7**  Method is complex or difficult to understand | | |
|  | **E4.8**  Other | | |
|  | **E4.9**  Limitations not described | | |
